# Parents' champions vs. vested interests: Who do parents believe about MMR? A qualitative study

**DOI:** 10.1186/1471-2458-7-42

**Published:** 2007-03-28

**Authors:** Shona Hilton, Mark Petticrew, Kate Hunt

**Affiliations:** 1 MRC Social and Public Health Sciences Unit, 4 Lilybank Gardens, Glasgow, UK

## Abstract

**Background:**

Despite the Government acting quickly to reassure parents about MMR safety following the publication of the 1998 paper by Wakefield and colleagues, MMR uptake declined. One of the reasons suggested for this decline is a loss of public trust in politicians and health professionals. The purpose of this analysis was to examine parents' views on the role the media, politicians and health professionals have played in providing credible evidence about MMR safety.

**Methods:**

A qualitative focus group study conducted with parents living in Central Scotland. Eighteen focus groups were conducted with 72 parents (64 mothers and 8 fathers) between November 2002 and March 2003. Purposive sampling was used to ensure maximum variation among parents.

**Results:**

In the period after the MMR controversy, parents found it difficult to know who to trust to offer balanced and accurate information. The general consensus was that politicians were untrustworthy in matters of health. The motives of primary health care providers were suspected by some parents, who saw them as having a range of vested interests (including financial incentives). Among the sources of evidence seen by some parents as more credible were other parents, and Andrew Wakefield who was viewed as an important whistle-blower and champion of ordinary parents.

**Conclusion:**

The provision of accurate information is only one aspect of helping parents make immunisation decisions. Establishing and maintaining trust in the information provided is also important. The MMR controversy may provide useful lessons for health professionals about trust and credibility that may be generalisable to future health controversies.

## Background

In Britain in October 1988, the combined measles, mumps, and rubella (MMR) vaccine was introduced into the routine childhood immunisation programme, replacing the monocomponent measles vaccine. By the early 1990s, MMR coverage for 2-year-old children exceeded 90% nationally and cases of measles fell to historically low levels [1]. In 1998 Dr. Andrew Wakefield and co-authors published a case series of 12 children in which they raised the possibility of a link between the MMR vaccine, autism and inflammatory bowel disease [2]. Despite the fact that the study received little support from the scientific community [3-5] and that the British Government acted quickly to reassure parents of the safety of the vaccine, these assertions led to a decline in MMR uptake. MMR coverage fell as low as 58% in some parts of the UK, and subsequent outbreaks of measles and mumps occurred [6,7]. Unlike many other health scares that are short-lived, stories about the safety of the MMR vaccine made headline news for some years, and there has been continuing interest in the issue from the media, politicians, health professionals, and parents alike.

A number of recent studies have explored how the controversy has been communicated to parents by health professionals and others, and their views of the reliability and trustworthiness of these sources of information. One qualitative study found that parents have not been convinced by the Department of Health's reassurances that MMR is the safest and best option, and it has been found that parents consider the information provided by health professionals to be biased [8]. For example they are aware that GPs need to reach immunisation targets and receive financial incentives to do so under the Childhood Immunisation Payments system [9]. Some GPs have criticised the immunisation payment system on the grounds that it increases parental distrust [10].

Attempts to achieve balanced reporting in the media may actually introduce bias, however unintentionally. In the UK, the presentation of both sides of the MMR debate in the media created the misleading impression that the evidence for the link with autism was as substantial as the evidence against [11]. It has also been suggested that the huge amount of media coverage has led parents to conclude that there must be some truth in the suggestion that MMR could be harmful [12]. However, communicating risk effectively to the public is about more than providing information [13]. The perceived trustworthiness of the sources providing information, including the Department of Health, and wider public scepticism about Government pronouncements on health following the BSE outbreak, also play a part [14].

Public distrust of health information may also have been heightened by Government's handling of the BSE, foot and mouth crises and salmonella and E. coli outbreaks, during which Government agencies responsible for food safety were perceived to have put the interests of food producers before those of consumers [15]. This growing public scepticism of the Government's trustworthiness in safeguarding public health provides the backdrop against which parents are expected to weigh the risks and benefits of MMR immunisation.

As well as raising issues about the credibility of organisations, the MMR controversy has been unusual in highlighting the contribution of specific individuals. For example, Burgess et al. refer to the role of the media in presenting Dr. Andrew Wakefield as "a lone voice against the establishment in a David and Goliath struggle"[14]. Wakefield remains in the public eye as a result of the General Medical Council decision in June 2006 to charge him with serious professional misconduct. Another key figure, Dr. Viera Scheibner, a public opponent of vaccination has toured North America, Europe and Australasia claiming that vaccines are ineffective and dangerous. These individuals have often been presented as "whistleblowers", or as having the best interests of parents and children at heart in apparent contrast to 'uncaring' scientists, and their positioning as 'independent' scientists may have made their views more credible to parents than those of GPs, epidemiologists or politicians who some assert, have their own vested interests [16].

Given this background, it is perhaps not surprising that it has been difficult to build public trust in MMR. Experts suggest the battle for public trust needs to be based on a better understanding of the nature of public concern [17], and that the trustworthiness of various sources of information appears to be key [18]. This study offers a unique insight into the debate about MMR and public trust by exploring opinions from an extensive range of parents and by explicitly examining the precise nature of their concerns about MMR safety.

## Methods

### Sampling and recruitment

Eighteen focus groups were conducted with parents living in Central Scotland between November 2002 and March 2003. Purposive sampling was used to obtain a diverse sample of parents in terms of age, socio-economic circumstances, likely views about vaccination, and family circumstances, including first-time mothers, more experienced mothers, single fathers, and parents with multiple social problems. The sample also included parents with a range of vaccine decision-making outcomes, including parents who had fully immunised, opted for single vaccines, rejected MMR, and rejected all vaccinations. Two additional groups were conducted with parents who had autistic children and with parents who had an immune-compromised child following chemotherapy (see Table 1). Following pilot work, the focus groups were held with small groups of between three and five people to allow each parent enough time to express their views and opinions and to facilitate later identification of individuals.

**Table 1:** Participants in the focus groups

**Group number and brief description**	**Participants' pseudonyms (ages of children, vaccine status)**
G1 NCT group in affluent area	Trudie (girl 8 yrs/girl 7 mths, both complete) Violet (girl 2 yrs, partial) Mel (boy 4 yrs, complete/girl 15 mths, partial)
G2 First time mothers	Joanne (boy 5 mths, complete) Elaine (girl 4 mths, complete) Louise (boy 5 mths, complete) Beathan (girl 6 mths, complete)
G3 Ante-natal group with second time mothers	Sian (stepson 4 yrs, other/8 mths pregnant) Dawn (boy 4 yrs/boy 3 yrs, both partial) Ruth (boy 1 yr, complete) Beatrice (boy 18 mths, partial) Iona (boy 12 yrs/girl 7 yrs, girl 5 yrs/boy 3 yrs, all complete)
G4 Low MMR uptake area in deprived area	Joan (girl 20 mths, complete) Sheila (girl 10 yrs/girl 3 yrs/boy 21 mths, all complete) Alan (boy 2 yrs, partial)
G5 High uptake area in affluent area	Fiona (girl 6 yrs/boy 5 mths, both complete) Alison (boy 15 mths, partial) Lauren (boy 14 mths, partial) Cassie (girl 3 yrs/boy 9 mths, both complete) Karen (girl 7 yrs/girl 4 yrs/boy 4 mths, all complete) Anna (girl 7 yrs, complete/boy 4 yrs, partial/girl 3 yrs, partial/girl 14 mths partial)
G6 Low MMR uptake area in deprived area	Cathy (girl 3 yrs/boy 2 yrs, both complete) Bob (boy 3 yrs/boy 7 mths, both complete) Ella (boy 5 yrs, partial) Helen (girl 4 yrs/boy 2 yrs, complete)
G7 High MMR uptake area in deprived area	Tracy (boy 10 yrs/girl 4 yrs/girl 2 yrs, all complete) Samantha (boy 16 mths, complete) Lydia (boy 6 yrs, complete) Angie (boy 5 yrs/boy 18 mths, both complete) Mary (girl 5 yrs, complete)
G8 Young single mothers living in deprived area	Kate (boy 2 yrs, complete) Margaret (boy 2 yrs, partial) Lisa (girl 23 mths, complete) Ann (boy 6 mths, complete) Lynne (boy 13 mths, complete) Natalie (boy 15 mths, complete) Ros (boy 20 mths, complete) Lucy (15 boy 11 wks, complete)
G9 First-time mothers living in affluent area	Rhona (boy 11 wks, complete) Catrina (girl 11 wks, complete) Judith (girl 11 wks, complete) Charlotte (girl 5 mths, complete) Celia (girl 6 mths, complete)
G10 Single fathers in deprived area	William (12 yrs,/girl 5 yrs, both complete) Kenny (boy 3 yrs other/boy 3 mths, complete) Robert (girl 17 yrs/boy 6 yrs, both complete)
G11 Parents with multiple parenting problems in deprived area	Sheena (girl 7 yrs/boy 6 yrs/boy 2 yrs, all complete) Michelle (boy 2 yrs/girl 6 mths, both complete) Patsy (twin boys 2 yrs both complete/boy 2 mths too young), Frank (as far Patsy)
G12 Single vaccine group (Parents who opted to immunise their child with separate measles, mumps and rubella vaccines)	Dave (girl 7 yrs complete/girl 21 mths, single) Jenny (boy 2 yrs, single) Joe (boy 2 yrs, single)
G13 Parents who had rejected MMR	Sue (boy 6 yrs, complete/boy 4 yrs, partial/boy 13 mths, complete), Aleena (girl 5 yrs/girl 3 yrs/girl 5 mths, partial) Hannah (boy 4 yrs/boy 2 yrs, partial)
G14 Parents who had rejected all immunisation	Molly (boy 5 yrs/boy 2 yrs) Kitty (boy 6 yrs/boy 4 yrs/boy 2 yrs) Lola (boy 6 yrs/boy 4 yrs/boy 2 yrs) Debbie (boy 5 yrs/boy 3 yrs/girl 23 mths/girl 4 wks) All none
G15 Parents of an autistic child	Lesley (complete) Dianna & Jacqueline (partial) The precise ages of children omitted to protect their identity -all boys aged between 4 and 7 yrs)
G16 Parents of an autistic child	Stella, Alison, Caroline (all partial) The precise ages of children omitted to protect their identity -all boys aged between 4 and 9 yrs)
G17 Parents of an immunocompromised child	Sally (girl 9 yrs) Rebecca (girl 8 yrs) Pamela (girl 8 yrs) all complete
G18 Parents of an immunocompromised child	Jill (girl 14 yrs), Cara (girl 8 years), Jessie (boy 16 yrs) all complete

Key:

*Complete*: parents whose children had received all the recommended vaccines for their age in the Childhood Immunisation Programme.

*Partial*: parents whose children who had fallen behind the recommended schedule with some vaccines, and parents who had decided not to have one or more of the recommended vaccines in the programme.

*Single*: Parents who had opted for the single measles, mumps or rubella vaccines instead of MMR.

*None*: Parents who did not plan to immunise their children with any of the recommended vaccines.

*Other*: Parents who did not know the immunisation status of their child.

### Data collection and analysis

A topic guide for the discussions was developed through pilot work. The guide included parents' understanding of the evidence about the safety of the MMR vaccine and their perceptions of the role that the media, politicians, and health professionals have played in the controversy. The discussions lasted between one and two hours and were facilitated by SH. To enable systematic comparisons to be made across the large amounts of data, each transcript was checked and imported into NVivo 2.0. Data were thematically coded and, following the principle of the constant comparative method, and rigorous analysis, [19] each transcript was repeatedly re-examined and cross-compared to identify common themes and explore parents' underlying reasoning. Once all the relevant extracts of data had been retrieved and checked we started to develop a coding frame around which to examine parents' concerns and views about MMR safety. Particular attention was paid to deviant or contradictory cases [20] and to group dynamics using field note observations [21]. One of the advantages of using focus group methods is that it can generate rich and dynamic data by encouraging discussion between group members. However the large amount of data generated has meant that it was neither feasible, nor desirable, to present all the main themes emerging from the data in one paper. Findings on the other two main themes, people's understandings of the vaccine-preventable diseases and parental concerns about "vaccination overload", have been reported elsewhere [22,23]. The sometimes chaotic nature of focus group conversation and the need to balance the group picture against the voices of individuals make reporting focus group data particularly challenging. To report the data in this paper we have mainly selected concise quotes attributable to an individual, but during analysis have been mindful of group effect and the fact that all conversation is influenced by the context in which it is generated [24]. Ethical approval for the study was obtained from the Glasgow University Ethics Committee for non clinical research involving human subjects.

## Results

Eighteen focus groups were conducted with a range of parents, including 64 mothers (age range 15 to 53 years, mean age 32 years), and eight fathers (age range 31 to 51 years, mean age 39 years). In relation to the credibility of sources of evidence or information on the risks of MMR, five main bodies were identified: other parents; the media; politicians; health care professionals; and Andrew Wakefield himself. These are each considered in turn.

### 1. Other parents as credible sources of information on the risks of MMR

The parents that took part in this study had a range of views about immunisation and had made different decisions about the MMR vaccine, but across all the groups the general perception was that the evidence of a causal link between MMR and autism was not convincing at present, but that in time further evidence could come to light to show that MMR is damaging for some children. In the meantime, participants felt that the evidence from the parents who believed that MMR harmed their child could not be discounted:

Trudie: *I just don't think enough research has been done really, one way or the other, to say whether it is completely safe*.

Mel: *I still feel as if there is something underlying, something there, you know these children were they *(interrupted)

Violet: *You know though that these parents weren't just making it up, I don't think, you know*.

Mel: *No, I know*.

Violet: *I think to say there is no evidence that it causes harm, is not comforting, because that just means there has not been the research done on it. You could say that about virtually anything practically*.

Trudie: *That's true*.

(G1 NCT affluent area group)

The stories of other parents were viewed as being more impartial as they were seen to have no "hidden agenda", and their stories were easy to relate to: *"...You know where you are with other parents. They don't have any reason to make things up or like any hidden agenda so to speak, so you feel you can believe other parents"*. (Patsy: G11 Parents with multiple parenting problems). In this respect these anecdotal accounts from other parents appeared to carry as much, if not more, weight than either evidence from epidemiological studies or assurances from politicians and public health officials. Parents could understand other parents' concerns and could assess their credibility. This was not the case with research studies, which many participants felt ill-equipped to assess for themselves.

### 2. The credibility of the media as a source of evidence

Some participants stated that they found it difficult to distance themselves from the debate, and (confirming the importance of other parents as credible sources of evidence) stated that they felt particularly drawn to newspaper stories that involved real life people. For example,

"... I think there's a sense that there's a kinship with other parents that you just don't have with, you know, doctors... And I think as well, you know, that the evidence that scientists use, it's just stuff that just goes in and out your ears. You just can't comprehend it. It's not written for parents, and then when they do write it for parents you just wonder, you know, what their motives are because there are so many big players, so many people with their own interests that it's easier to believe other parents. You want to believe other parents."

(Dave: G12 Single vaccine group)

Nonetheless parents' views on the role of the media varied widely. Some viewed journalists as scaremongers, whilst others thought of them as valuable information providers. For example, one father considered that: "*...the newspapers are trying to let the everyday people know the inside story*" (Frank: G11 Parents with multiple parenting problems). However, it was more common for parents to speak negatively about the media's involvement in the MMR debate. One mother stated angrily that: *"...the media have a responsibility to stop just taking bits of research and throwing it into the press to alarm us*" (Iona: G3 Ante-natal group). Parents also felt that health stories, especially those involving children, are of huge interest to the general public and that the media are acutely aware of this fact. A few parents complained that the media presented the evidence in such a way that it was difficult to derive clear messages about the safety of MMR. In particular, they criticized the tendency to place scientific and anecdotal evidence alongside each other, in an attempt to create balance, but in reality this left some parents confused. The high level of media attention paid to the debate also appeared to have influenced parents' assessment of the evidence. It was implied on several occasions that the fact that so much attention had been afforded to MMR was on its own evidence that MMR is unsafe: "*...there's no smoke without fire*" (Margaret: G8 Young single mothers group).

### 3. The credibility of politicians as a source of evidence

The general consensus among parents was that politicians were untrustworthy in matters of health. Parents recalled the previous government's handling of the BSE crisis in the 1990s when they felt that the public had been misinformed. One particular similarity was mentioned; the role of politicians' own children. The image of John Gummer, a former Minister of Agriculture, feeding his daughter a hamburger in 1990 to show that British beef was safe was mentioned by parents as symbolising the Government's handling of the BSE crisis. Parents drew a parallel with UK Prime Minister Tony Blair refusing to confirm in 2001 whether his baby son Leo had had MMR. This was discussed within many of the groups and parents often debated at length the rights and wrongs of Blair's decision not to disclose this information. For example, one mother considered: "*I don't really think it is an issue of the baby's privacy, either he has had it, or not... He should come out and say*" (Molly: G14 Parents who had rejected immunisation group). A father agreed: "*The fact that he didn't disclose that information has put fear into parents... He may be pushing a programme that he doesn't believe in*" (Kenny: G10 Single fathers group). For others, however the pressure to immunise perhaps suggested "nanny-state" politics: *"... It's like a metaphor for the way the government treats the public. 'I know what's best for you – have a burger', sort of thing*" (Sue: G13 Parents who had rejected MMR). Parents were often dismissive of phrases such as 'no proven risk,' and 'minimal risk,' and of official messages that MMR is safe, and appeared to interpret such assurances of vaccine safety as meaning that experts are not aware of any risk 'at the moment'. For example, one woman said: *"throwing blanket statements at you, it's safe, there's no proven risk just doesn't reassure you... it reeks of all the other health scare scandals. Where we were told, there is not a problem, not a problem- oh whoop! There is a problem." *(Dawn: G3 Ante-natal group with second time mothers). The general view expressed by parents was that politicians serve their own and their party's interests before that of the public.

### 4. Health care professionals as sources of evidence on MMR

Parents' views on the role that health professionals were felt to have played were mixed and highlighted some concerns about the objectivity of GPs and others. The dilemma that many parents appeared to face was one of knowing who to trust to give them impartial advice. One mother of a boy with autism asked:

"What do you do as a parent? You don't know who to trust. Because these are the people- you're meant to trust your doctor implicitly and yet people are saying well, you know, they're getting paid for having so many people vaccinated and all this, and you start thinking 'well... who's got my wee boy's best interests at heart' "

(Lesley: G15 Parents of a child with autism)

Similarly, another mother questioned the extent to which parents can rely on health professionals to give them impartial advice. She said that she felt: "*...suspicious of some of them, I just sort of don't know their motives, so you know, that does concern me, because you know is there profit involved in it?*" (Helen: G6 Low MMR uptake area group). Central to this dilemma seemed to be parents' increased awareness that GPs receive payments for meeting Government immunisation targets. A common theme was that parents did not know to what extent their own GP or health visitor was acting in their child's best interest, as opposed to acting in their role as an advocate of public health policy. As one mother put it: "*they are part of the system of dispensing it; they're not there to question*." (Sue: G13 Parents who had rejected MMR).

As for health visitors, when they sounded too resolute about the safety of MMR, some parents questioned their motives and knowledge; conversely when they sounded more vague, some parents interpreted this as concern that MMR is unsafe. Several of the parents who had either decided to delay, or opted not to have MMR, spoke of their health visitors applying unwanted pressure and in some cases ostracising them for not complying with the recommended vaccines. Some of the parents who had opted to have single measles, mumps and rubella vaccines, talked about feeling 'blackballed' from their surgery (Jenny: G12 Single vaccine group).

### 5. "That doctor..." Andrew Wakefield as a credible source

While parents often spoke of concern about their own doctor's presumed lack of impartiality, one particular doctor at least was seen by some as an important and credible source of information. For some, Andrew Wakefield was an important whistle-blower and champion of ordinary parents. More importantly he was perceived by some to provide the necessary balance which they felt was often missing from other accounts: *"...at least Dr Wakefield has stirred things up and got people asking questions" *(Stella: G16 Parents of a child with autism). Criticism of Wakefield by public health officials appeared counter-productive, and if anything, was taken as evidence of their attempts to suppress the 'truth': *"I just think the government lie about everything... and try to discredit the doctors...you know, Wakefield*" (Angie: G7 High MMR uptake area group); *"...instead of saying 'no, no, not possible', they should take Dr. Wakefield's work seriously" *(Dawn: G3 Ante-natal group). For some, Andrew Wakefield represented the voice of reason:*" this doctor who has had all these parents coming to him has said, you know look, I'm not saying that it is a cause, but there is enough concern to be worried about it" *(Joanne: G2 First time mothers group).

Not all parents agreed with this analysis. Some implied that Wakefield should shoulder much of the blame for their uncertainty about MMR safety:

*"See, really, afore this all came out, that doctor, that doctor should have had their facts perfect, the facts that they should have been right before they came away out with all this. It just seems as if they've blew it all out of proportion and then they retract some of it"*. (Alan: G4 Low MMR uptake area).

## Discussion

These findings identify some of the problems which parents perceive in identifying reliable sources of information on MMR risks and benefits, and in balancing these to make decisions about immunisation. Some viewed the media as pivotal players in much of the unsettling dialogue played out in news reports in newspapers and on the television, consistent with Clements and Ratzan's suggestion that the media have been alarmist [13]. In contrast, other parents viewed the media as valuable sources of evidence and seemed unquestioning about some of the stories they heard and read. They also appeared to be under the impression that there was as much evidence showing a link, as showing no link, consistent with the findings of the media analysis carried out by Hargreaves et al. (2003). Parents spoke of feeling particularly drawn to anecdotal stories involving real people, and spoke about finding other parents' stories more convincing than statistics and reassurances from scientists and politicians, perhaps because, unlike doctors, they were perceived to have no apparent conflicts of interest – only, quite naturally, the interests of their own children. This suggests that for public health communicators it may be better to make judicial use of parents themselves to help communicate with the public about MMR – and to use them not just as providers of factual information on vaccine safety, but as a way of explicitly acknowledging the difficulties and dilemmas parents face.

Importantly, many parents appeared not to know whom to trust to give impartial advice, and some were suspicious of their GPs' motives. Whether parents trusted the information health visitors gave depended on how well-informed and open to debate they appeared to be. Conversely, ambiguous or entrenched views made parents less trusting of the advice. Perhaps for this reason Andrew Wakefield's apparent willingness to debate the issue of vaccine safety made him seem more open and therefore trustworthy to parents, than doctors who sounded too resolute about MMR safety. Indeed, any attempts to criticise him and his research only appeared to serve to emphasise his credentials as a "defender" of the rights and concerns of ordinary parents. It is perhaps surprising for one individual scientist or doctor to feature so strongly in public accounts of a scientific controversy. The data presented in this paper were collected before Wakefield's findings became widely discredited, and before ten of his co-authors published a retraction in the Lancet in 2004 [25]. Wakefield has however been placed back in the media eye as a result of the General Medical Council investigation [26] and if our research were to be repeated it would be interesting to see whether his perceived trustworthiness has been sustained.

A major strength of the study is that it includes an extensive sample of parents representing a wide range of potential views on the MMR debate. This sampling strategy is particularly useful when the aims is to gain an 'in-depth understanding', rather than 'overall picture', of a topic. Thus the sample is not intended to be statistically representative and includes a higher proportion of vaccine sceptics in order to gain new insights into parents' concerns about the MMR debate. It is anticipated that the findings in this paper will usefully contribute to the wider debate about trust, credibility, conflict of interest, and to understanding how to regain parental confidence in childhood immunisation. However among the limitations is the fact that the MMR debate is continuing to develop and unfold, and parents' views may change in the light of new research, new campaigns and new media coverage. There are also specific features which may make the MMR controversy unusual, not least the emphasis on the role of Wakefield as a source of evidence, and as a "parents' champion". Nonetheless our findings bear out the general conclusions of Brownlie and Howson (2005), that in the case of MMR, trust in information providers is not just about the quality of the information provided, but is also about: "the wider socio-political changes...which parents, professionals and indeed health authorities have to negotiate"[17].

There are several implications for future research and practice. The first relates to parents' perceptions of the vested interests of health care professionals. Although parents' satisfaction with health professionals with respect to immunisation visits is high [27], some are suspicious of the motives of GPs and health visitors, in part because of their awareness of the Immunisation Target Payment system. Both GPs and the Department of Health need to be aware that patients' awareness of these fees may compromise their value as sources of objective advice in the eyes of parents, and the scheme may be potentially damaging to the parent-practitioner relationship and to the achievement of vaccination targets. This may mean either that such payments compromise trust to such an extent that their use should be abandoned, or that communicators should directly acknowledge that they exist and explain their purpose.

A related point is that it is generally difficult for parents to know who to trust to offer balanced and accurate information on which they can make sound judgments. This has general lessons for future education campaigns on immunisation, which may require greater efforts to ensure that patients are presented with information that is seen to be reliable and unbiased. On the basis of the evidence presented in this paper, the potential downside of this is that what health professionals see as "unbiased information" may sometimes be viewed as unhelpful and non-directive, or at worst reflecting a conflict of interest. This suggests that in future campaigns risks, benefits and conflicts need to be acknowledged clearly and openly, and that, as acknowledged above, apparent conflicts of interest should be addressed or explained where it is anticipated that these will reduce public trust. This includes highlighting to the public the financial conflicts of interest which sometimes underlie the criticisms of apparently independent scientific experts and critics.

Development of future effective communication strategies may also usefully be informed by considering Lupton's perspectives on risk and trust [28]. She suggests that when people perceive 'expert knowledge' to be failing, they turn to local knowledge to're-embed' their trust in those whom they know personally. There was some evidence that parents sought guidance from local knowledge by seeking advice from their health visitors. However because health visitors themselves seemed ambiguous about the safety of MMR, parents questioned the extent to which they could trust their advice. It is possible that some health visitors were unable to reassure parents because they themselves felt inadequately informed about new developments in the MMR debate. The failure to reassure parents may also reflect a wider a disparity between the expectations parents and health visitors have of the role health visitors play in childhood immunisation. What is needed is an examination of health visitors' views of their role in the MMR controversy in order to develop better guidance for communicating public health messages to parents during times of uncertainty. A stronger emphasis on building trust than on providing more, or better information may be needed [17]. Building trust involves health professionals becoming engaged in more open dialogue which focuses attention on addressing parents' individual concerns rather than on just providing factual information about immunisation.

The issue of lack of trust may also be addressed by directly confronting the issue of independence. The public may value the opinions of MMR critics specifically because they appear to offer independent, non-aligned expert opinion, though they may not appreciate that these experts too may be subject to conflicts of interest. Perceived independence of professionals is known to be important to the public in other spheres of risk communication [29], and in general doctors continue to be considered to be independent and trustworthy [30]; anything that compromises independence therefore undermines their role as communicators of risk. Miller and Macintyre note that risk communication is not a one-off event, but a continuing dialogue; and so although MMR coverage is increasing, the need to ensure that communication with the public about risks of vaccines and to build trust in professionals remains. While "rebuilding trust" may seem a somewhat paternalistic goal, it can also be consistent with active patient involvement, and with greater concordance [31].

## Conclusion

The MMR debate continues to raise important and wide-ranging issues in relation to perceived conflicts of interest, and the lack of trust in providers of health information. The General Medical Council's decision to formally discipline Dr Wakefield in 2007 may bring about some sort of resolution to the MMR debate. In the long term this controversy may provide wider lessons for health professionals and governments about trust, credibility and risk communication- lessons which may be applied to as yet unknown health crises and controversies.

## Competing interests

The authors declare that they have no competing interests. The Medical Research Council (MRC) funded Shona Hilton's PhD studentship, as part of which these data were collected. KH is funded by the MRC. MP is funded by the Chief Scientist Office of the Scottish Executive Department of Health.

## Authors' contributions

SH, MP, KH participated in the design of the study. SH coordinated, collected and analysed the data. All authors contributed to the drafting, writing and redrafting the final version of the manuscript and have approved the final manuscript.

## Pre-publication history

The pre-publication history for this paper can be accessed here:


